# Hôpital de la Pitie

**Published:** 1829-01-01

**Authors:** 


### HOPITAL DE LA PITIE.

I. On the Treatment of Cancerous Affections of the Cervix Uteri, and especially on Amputation of that Part. By M. Lisfranc. Reported by M. Avenil.

In a late number of our Parisian contemporary, [The Revue Medicale] there is a long Memoir on this important subject, and ten cases are detailed of this formidable operation. We shall, as usual, greatly compress the matter of the Memoir before us.

M. Lisfranc commences with some observations on the accidents which may or do supervene on the operation of excision. The first of these is hæmorrhage. This accident, he thinks, is too much dreaded by surgeons, and the pluggings of the vagina, to which they have recourse for its suppression, leads to inflammation and worse consequences than the hæmorrhage. On these accounts, he always permits a copious flow of blood from the excised parts.

"When a woman has not been exhausted by preceding bleedings—when she is not extremely weak—when the hæmorrhage does not appear to proceed from large vessels, but, on the contrary, is so slow that there is not more than twelve or fourteen ounces lost in the first two or three hours,—then the hæmorrhage should not cause any alarm in the surgeon's mind—nor should the process of plugging the vagina be employed."

M. Lisfranc here describes a series of phenomena which take place when a clot of blood forms in the passage, and produces the same effects as plugging. Among these are, pain in the loins, epigastrium, and region of the uterus, sense of distention, hiccup, nausea, vomiting. When this last occurs, the clot is generally driven away, and all the symptoms are immediately relieved. When the above-mentioned phenomena, however, continue, without this natural relief, M. Lisfranc introduces his fore-finger, breaks away the coagulum, and re-excites the hæmorrhage, when the strength of the pulse, and the state of the patient appear to warrant the renewed loss. When the mechanical removal of the clot does not re-produce the hæmorrhage, he throws up warm water, which generally has the desired effect. After the storm has ceased, it often happens, that, two or three times in the course of the day, sickness and vomiting recur. On this account, the patients in La Pitié are always operated on at nine o'clock in the morning, and all the troublesome symptoms are usually over by five or six in the afternoon. When the spontaneous, or artificially excited hæmorrhage does not relieve the symptoms, M. Lisfranc prefers bleeding from the arm to leeching the groins or labia. He thinks the former acts more beneficially than the latter. Low diet, diluents, lavements, emollient fomentations, &c. are had recourse to, as auxiliaries, if the foregoing measures fail. Generally a most delightful calm succeeds the first effects of the operation. The dreadful disease removed, the patient tastes a refreshing sleep the first night, to which she had been long a stranger. There is a marked change in the morale as well as in the physique. Her gaiety is renewed, and her gratitude to the man who has delivered her from such torments, is expressed with unbounded energy. A complete convalescence is often observed in two or three days after the operation. But great care, absolute repose, and low diet, should be insisted on for eight or ten days, even where no bad symptom appears.

In two cases, where nothing particular occurred during the first two or three days, and where there was no appearance of hysteritis or peritonitis, nor any sign of them on dissection, the patients were suddenly seized with pain in the head, dizziness, weakness, and quickness of pulse, great prostration of strength—death. In these cases, M. Lisfranc asks if the fatal issue did not depend on the absorption of cancerous or poisonous matter from the wound, as there was nothing, on dissection, to account for their death?

When the cicatrization of the wound does not proceed in a favourable manner, M. Lisfranc employs injections containing solutions of the chloride of soda, weak at first, and gradually increased in strength. If this fails, and fungous excrescences are detected on the surface of the wound, he employs the cautery. The medium period required for cicatrisation, is six weeks or two months. Great attention should be paid to the general health for a long time after recovery.

CASES.

*Case* 1. Constantia Franck, aged 33 years, became affected with pains in the uterine region, after great mental anxiety and reverses of fortune. Towards the close of 1826, she left Brussels, and came to Paris to consult M. Lisfranc. He detected an incipient cancer of the cervix uteri; and some ulcerations had already taken place around the os uteri. The pains were lancinating and constant, with fetid excoriating discharge. Two months of antiphlogistic measures produced no amelioration of the symptoms, and she entered LA PITIÉ to undergo the operation, on the 3d of January, 1827. Three weeks had elapsed since the last consultation, and the disease was found to have made progress. The operation was performed, according to M. Lisfranc's usual method (already described in this Journal) and the patient suffered little. In one hour after the operation, the hæmorrhage became very abundant. M. Lisfranc sat by the bed-side, and did not plug the vagina. In two hours, the bleeding lessened, and a clot formed. Colicky pains next occurred, and a fit of vomiting expelled the clot. The pains immediately ceased. A slight syncope supervened, but the pulse did not sink much, and the plugging was avoided. In the same evening, there were symptoms of excitement that rose into strong re-action, requiring venesection to six ounces. We need not pursue the diurnal details. The nitrate of silver was applied on the 29th of January. On the 24th February, the patient left the hospital cured.

*Case* 2. Ellen Loos, aged 30, the mother of six children, became affected, after two abortions, with lancinating pains towards the groins, loins, thighs, and region of the uterus. The menses got irregular, and, for nine months previous to her entrance into LA PITIÉ, she had considerable discharge from the vagina. Her mother died of cancer of the uterus. The patient entered the hospital on the 20th January, 1827. She had had 300 leeches applied in the course of three months at two other hospitals. On examination, the cervix uteri presented considerable tumefaction, and deep ulcerations, from which a fetid sanious pus issued. The operation was deemed indispensible, and was performed on the 23d of the same month. The uterus, in this case, could not be drawn as low down as usual, although it was in a state of slight prolapsus. Five lines in depth of the cervix uteri was removed. Considerable hæmorrhage ensued, and most of the symptoms described in the preamble of this paper. But the patient ultimately did well, and, by the 18th of March, the cure was complete.

*Case* 3. Augustine Conder, aged 25, became affected with lancinating pains in the uterine region shortly after an abortion, which she had experienced eight months before she entered the hospital, which was on the 15th February, 1827. The cervix uteri presented a large ulceration, especially on the anterior lip of the os uteri, with reverted edges, and an indurated state of the neighbouring parts. The uterus itself was double its natural size. The operation was performed on the 19th, and offered nothing worthy of remark. Very little pain was experienced while the cervix was incised—the whole of the diseased part was removed—and there was scarcely any blood lost. Some smart hæmorrhages afterwards supervened, but they did not require the plug. There were considerable pains, and symptoms of constitutional disturbance in the course of the first few days, requiring several bleedings, and all the usual antiphlogistic measures. On the 1st of March, the menses appeared, and flowed for two days, and on the 14th of the same month, she left the hospital completely cured.

*Case* 4. Harriet Cuiret, aged 22, had never borne children. Had experienced, for more than a year, lancinating pains in the region of the uterus, with an abundant leucorrhœa. The menses became irregular, and, for the three preceding months, had scarcely been seen. She entered LA PITIÉ on the 30th of January, 1827, for another complaint, during the cure of which, she disclosed the more important malady. The cervix uteri was found to be tumefied, indurated, profoundly ulcerated. The operation was performed on the 5th of March, as M. Lisfranc was of opinion that there was no time to lose. There was great difficulty experienced in the resection, on account of the narrowness of the parts. The surgeon was obliged to use considerable force in separating the parietes of the vagina, and perform the section of the cervix in the interior of the vulvo-uterine canal. There was little blood lost at the time; but great hæmorrhage succeeded, and was arrested by syncope and the formation of a clot. The clot was afterwards expelled by vomiting—and these formations and expulsions were four or five times reiterated alternately. In one of these hæmorrhages there was a loss of more than 20 ounces from the wound. On the 7th April, a copious menstruation took place; and on the 11th of the same month, she was seized with an intermittent fever, which ceded to the quinine. On the 27th July, she was discharged perfectly cured.

*Case* 5. Claudine Deschamps, aged 35, was delivered safely of a child in December, 1826, soon after which, the menses were suddenly suppressed by a violent mental emotion. Uterine pains, of a lancinating character, next succeeded, and, on the 6th March, 1827, she entered LA PITIÉ. Large vegetations were discovered on the cervix uteri, and similar excrescences were seen about the verge of the anus. M. Lisfranc thought them venereal, and prescribed mercury, under the influence of which, the external vegetations completely disappeared; but the others continued unaltered. The cervix uteri now became tumefied, indurated, and, in fact, the malady made such progress, that M. Lisfranc decided on an operation. It was performed on the 28th May, at nine in the morning. The hæmorrhage was trifling till she was carried to bed, when it became considerable, and syncope was twice imminent, accompanied by chills. A large clot formed in vagina. At eleven o'clock that night, nausea, hiccup, vomitings, came on, and the clot was instantly expelled; when all the foregoing symptoms ceased. They again recurred, on the formation of a clot, and again the clot was expelled. There were some difficulties in the management of the case, but they were overcome; and, on the 30th June, a profuse menstruation came on. In five weeks the cicatrisation was completed.

*Case* 6. Madam R——, 42 years, perceived, in the year 1823, after some violent moral afflictions, severe pains in the loins and groins, accompanied by leucorrhœa, for which she underwent remedial treatment, without relief, for two years She then consulted M. Serres in Paris, who found the os tincæ surrounded with slight ulcerations, which were touched with caustic, and this process produced much relief. Eighteen months afterwards, she was induced again to return to Paris, and M. Serres found that extensive ulcerations obtained around the mouth of the uterus. He proposed ablation of the cervix, and M. Lisfranc was summoned. This gentleman found deep ulcerations, surrounded with stony hardness, and attended with fetid discharge. On the 26th April, the operation was performed, without any thing particular occurring afterwards; and on the 27th June, she was pronounced to be completely cured.

*Case* 7. Rose Rudell, aged 27, had experienced, for the five preceding years, and without any known cause, severe pains in the loins, copious leucorrhœa, shooting pains in the region of the uterus, irregular menstruation. These symptoms were accompanied by general derangement of the health, and a yellowish tint of the skin. She entered the HOSPICE DE PERFECTIONNEMENT in the beginning of October, 1827, at the time when M. Lisfranc was on duty there. On examination, by means of the speculum, he found ulcerations and induration of the cervix. On the 19th November, the cervix was completely removed, and it was evident from the ulcerated state of the parts, that the operation was not practised an hour too soon. The usual symptoms occurred after the operation, and she left the hospital completely cured on the 31st of December.

We do not deem it necessary to pursue the analysis of the remaining cases.

Sufficient have been adduced to shew the ordinary symptoms that precede the operation, and the accidents that follow it. In the course of the last four years, M. Lisfranc has performed this formidable operation, in the hospital and private practice, thirty-six times. In 33 cases the operation was crowned with success. Three of the patients died :—one on the 18th day after the operation—apparently from an affection of the liver, and an encephaloid disease behind the uterus—the second, after three months of good health, when the uterine disease returned—the third died from an affection of the spleen, not suspected to exist at the time of theoperation The reporter, M. Avenil, witnessed three other operations of this kind in Paris—one by Dr. Ricord, one by Professor Recamier, and one by Dr. Hutin. In two of these three cases complete success followed, and the patients now enjoy excellent health. One of them became pregnant in the course of a month after the operation, and was safely delivered. She has, since that period, borne twins.

When we consider the fatal nature of this disease—the dreadful and lingering torments by which the patients are consumed—the comparative facility of *this* operation, and the almost insuperable objections to a total ablation of the uterus, we cannot but regret that English surgeons should be behind their Continental brethren in courageously affording relief to their suffering country-women, upon such trying occasions. We hope the present Memoir will induce them to take early means for ascertaining the approaches of uterine disease, and that the success of M. Lisfranc and others, will stimulate them to operate before the progress of the malady renders the operation terrible, and the result precarious.

### II. TREATMENT OF VARICOSE ULCERS.

*Case* 1.—*Division of the Vena Saphena.*—A soldier, 32 years of age, of a good constitution, received a burn in the lower part of the leg, nearly two years before his reception in La Pitié. At this time there was an ulcer, the size of the palm of the hand, and several ramifications of varicose veins were perceived upon the leg. The vena saphena was divided by M. Lisfranc, below the knee; no symptom whatever of phlebitis succeeded, and in eighteen days, the wound had healed. In twenty-four days after the operation, the patient was discharged completely cured. He now returned to his usual occupations, retaining no bandage or compression on the leg, the cicatrix got abraded, and the patient re-entered La Pitié on the 1st of August. The ulcer was now not so large as before, and a subcutaneous vein, about as thick as a crow-quill had appeared before and below the cicatrix made by the former operation. Simple applications and the horizontal posture, effected a cure in eighteen days, when the patient was a second time dismissed, with careful injunctions to wear a laced stocking.

*Case* 2. *Ulcer of the Leg.*—*Excision of part of the Vena Saphena.*—Charles Perlot, ætatis 52, a house-painter, of only an indifferent constitution, was admitted into La Pitié, with an ulcer of twelve months' standing, a little above the internal malleolus. The sore was as large as the palm of the hand, and the treatment at first consisted in the application of emollients and poultices. On the 23d of June, a fortnight after his admission, part of the saphena interna was excised below the knee. On the day of the operation, he required a bleeding from the arm, and, complaining on the morrow of pain in the limb, sixty leeches were applied on the inside of the thigh, followed, on the 25th, by a relay of forty more. On the 26th, there was a third application of leeches, and the saphena, on the 27th, presented the symptoms of inflammation of its coats, viz. tension, swelling, and tenderness on pressure. Fifty leeches were placed on the upper part of the thigh, but these not sufficing, the same number was twice repeated. The symptoms of phlebitic inflammation subsided, an abscess formed about the middle of the thigh, it was opened, and a wine-glass full of pus escaped. From this time, no untoward accident occurred; the wound was healed at the end of five weeks, but the ulcer continued open for about two months, when a laced stocking was applied, and the patient dismissed.

In the first of these cases, *division* of the vein was had recourse to, in the second *excision*, and mark the results. In the former, no local irritation was excited; in the latter, phlebitis was the consequence, and the patient had a narrow escape with his life. Division, with a valvular opening in the skin, is, generally speaking, a safe operation; excision, or ligature, certainly is not. It is but the other day since a patient was lost at Bartholomew's Hospital, by cutting out a portion of a varicose vein, and the experience of the leading men in the profession is almost universally opposed to the practice. The opinions of Sir Astley Cooper, on the subject, are expressed in his usual frank and energetic manner.

"It was formerly the practice, when the veins were in a varicose state, to tie and divide them. This plan is still pursued by many surgeons; but it is one that I have deprecated in my Lectures, in this Theatre, for the last eight or nine years; it is very injudicious, and fraught with great danger, therefore, let me exhort you never to adopt it. I have seen this operation prove fatal in several instances, in these hospitals; therefore, I was induced to say, that it must not be performed. A gentleman, at Nottingham, informed me, that he had tied the vena saphena, for a varicose state of the veins of the leg of a young farmer, in other respects healthy, and the operation proved fatal. The same lamentable catastrophe occurred to a most respectable practitioner, at Brentford; and this gentleman told me, that he would not again perform the operation for the world. If I were to tell you all the cases in which I have known it terminate fatally, I should recount, at least, eight. Another overwhelming objection to the operation is, that, when it does not prove fatal, its ultimate effects are useless. If I were asked, which of the following operations I would rather have performed upon myself, viz. the saphena major vein, or the femoral artery tied, *I certainly should choose the latter*. When an artery is tied, the inflammation is confined to the neighbourhood of the ligature; but in a vein, it is very extensive, the vessel becomes exceedingly distended, the inflammation uncommonly severe, either extensive suppuration or mortification ensue, and death is the result."—*Sir A. Cooper's Lect. by Tyrrel, Vol.* 1.

The worst of it is, that even where the operation does not kill, it often fails to cure. Again and again have we witnessed division of the vein, followed by relief, indeed, a cure, for the time, but quite ineffectual in its ultimate results. The patient returns with the leg as bad as ever, other veins having arisen to occupy the place, or, we should say, the office of the one that is obliterated by the knife. Is the surgeon to continue cutting vessel after vessel, running risk after risk, or trust to compression, and palliate what it is not in his power to prevent? We should decide in favour of the latter proposition, and apply the laced stocking, or equable bandaging. Bandages, we think, are the better of the two, but seldom, in the upper classes, admit of being worn. The patent silk bandage (we really are ignorant of its exact *scientific* nomenclature) is at present much in fashion, and may be had at a mercer's in Oxford-street. The fact, however, is that its great elasticity, for which it has been praised, is its worst and most objectionable quality, as it *gives* far too much to make steady compression. This, in connexion with its comparatively high price, will for ever prevent its coming into general use. The lower classes cannot afford it; for the higher, it has most of the inconveniences, and not all the good properties of the cotton roller.

The ulcers which depend upon varicose veins, or varicose ulcers, as they are frequently, and not very properly called, are generally healed by repose, common stimulating applications, as the solution of the nitrate of silver, &c. and especially bandaging and strapping. We say they are healed, for a pellicle of skin will form upon the surface, in nine cases out of ten, by the above means. Though covered, however, with ashes, and extinguished to the view, the fire is lurking and smouldering below, ready to burst forth on the least exciting cause, and rage as extensively and fiercely as before. It is really astonishing how quickly these varicose ulcers will skin over, and how quickly again they will break out afresh, when the patient resumes his usual avocations. Careful and continued bandaging, with standing about as little as possible, are all the prophylactics that the surgeon possesses, or the patient can trust to. We shall not advert to the employment of blisters and irritating applications in the neighbourhood of the varicose vessels. They may be regarded rather as matters of curiosity than practical utility.

### III. Paraplegia.

It appears that this disease has been so frequent during the last year or two, in Paris, that M. Bally, the physician to La Pitié, considers it as exhibiting a kind of epidemic character. It appears, also, that M. Bally has been eminently successful in the cure of the disease, by means of strychnine, which he has exhibited, in some instances, to the extent of three grains per diem, beginning with an eighth of a grain, assisted by the application of numerous moxas to the spinal column. Dr. B. thinks that the disease is attributable to some atmospheric constitution, the nature of which is, at present, unknown. The disease is distinguished by a sense of numbness and tingling in the hands and feet, accompanied by a morbid sensibility of the surface, and an almost total loss of voluntary motion. But, although many have been cured, some have died; and of these last, two cases have been recently reported from the wards of La Pitié, of which we shall now proceed to give some account.

*Case* 1. Claveron, aged 27 years, entered the hospital on the 3d of May. This young man, during a residence of three years in Paris, where he had worked as a mason, was two or three times affected with dull pain in the loins, which obliged him to give up work for several days at each attack. Towards the end of January, 1828, these pains acquired a greater degree of intensity than usual, and compelled him to go into the Hôtel Dieu, from whence he was discharged, relieved, but incapable of pursuing his avocations. He next sought admittance into La Pitié, where repose, warm baths, narcotics, especially the acetate of morphine, were prescribed. On the 12th of June, he presented the following symptoms:—violent pains in the loins, accompanied by an œdematous swelling of the lumbar region, and lower extremities—extreme pain on the least motion being communicated to the above-mentioned parts—constant position on the back—paleness of the face—emaciation—sunkenness of the eyes—peculiar air of sadness and melancholy in the countenance, which led to the suspicion of masturbation. On strict inquiry, the patient confessed his addiction to this vice. To the foregoing symptoms were added cough and mucous expectoration, bad sleep, fever, nocturnal perspirations, occasional diarrhœa. Moxas were applied to the lumbar region, and external irritation afterwards kept up, together with the application of strychnine to the excoriated parts. The same was administered internally, as also the acetate of morphine; but the disease made progress, and, by the beginning of July, the paraplegia was complete, and general dropsy followed. On the 26th of the same month he died.

*Dissection.* There was evidence of peritoneal inflammation—the substance of the kidneys was altered, and they were strongly adherent to the surrounding parts. Their surface was red and highly inflamed. The thoracic organs were sound—brain pale—no water in the ventricles. On opening the spinal canal, there was some infiltration along the posterior surface of the spinal marrow. From the 7th dorsal to the last lumbar vertebra, there was observed between the bony canal and the dura mater, a layer of a kind of cheesy matter, of tuberculous appearance, but without any appreciable lesion of the spinal marrow itself. Thus the paraplegia, and the disturbance of function in those organs deriving their nerves from the spinal marrow, appear to have been occasioned by inflammation of the coverings of the medulla spinalis, and by the compression exercised by the layer of cheesy matter above described.

*Case* 2. Berlemot, aged 24 years, had given himself up to excesses in venery, during the Winter of 1827—8; and, towards the end of February, began to feel debility of the lower extremities, with occasional sense of formication, and numbness of the feet, which symptoms were generally relieved by repose. In the month of May, and after some venereal debauches, the foregoing phenomena recurred with increased violence, accompanied by neuralgic pains in the loins, and utter impossibility of moving the lower extremities. By rest, warm baths, &c. these symptoms were partly removed; but as the paraplegia became ultimately predominant, he entered La Cha-

RITÉ on the 3d of June, 1828. Besides the paralysis, the patient complained of painful startings of the lower extremities—sense of stricture rising from the loins to the thorax, and impeding respiration. Defæcation became very difficult, and there was incontinence of urine. An eschar being formed in the loins, strychnine was introduced by that channel, to the amount, at least, of two grains daily. There was some improvement at first—but gangrenous ulcerations formed on the hips, and suspended the administration of the strychnine, which seemed to be doing considerable service. He died on the 26th July, 1828.

*Dissection.* The membranes of the brain were injected, and the substance a good deal softened. There was a deposition of a cerebriform matter over a considerable extent of the spinal marrow, and opposite this deposition the medulla spinalis was softened and partially disorganized.

These two cases shew, that the disease is not to be remedied by any medicine after a certain degree of disorganization has obtained. The dissections would lead us to think, however, that, at an earlier period or stage of the complaint, much might have been done by energetic means applied to the spine and head.—*Clinique.*

